# Protective effects of taurine against muscle damage induced by diquat in 35 days weaned piglets

**DOI:** 10.1186/s40104-020-00463-0

**Published:** 2020-06-03

**Authors:** Chaoyue Wen, Fengna Li, Qiuping Guo, Lingyu Zhang, Yehui Duan, Wenlong Wang, Jianzhong Li, Shanping He, Wen Chen, Yulong Yin

**Affiliations:** 1 Hunan Provincial Key Laboratory of Animal Nutritional Physiology and Metabolic Process, Changsha, 410125 China; 2 Key Laboratory of Agro-ecological Processes in Subtropical Region, Institute of Subtropical Agriculture, Chinese Academy of Sciences, Changsha, 410125 China; 3 Hunan Provincial Engineering Research Center for Healthy Livestock and Poultry Production, Changsha, 410125 China; 4 National Engineering Laboratory for Pollution Control and Waste Utilization in Livestock and Poultry Production, Changsha, 410125 China; 5 Scientific Observing and Experimental Station of Animal Nutrition and Feed Science in South-Central, Ministry of Agriculture, Changsha, 410125 China; 6 Laboratory of Animal Nutrition and Human Health, Hunan Normal University, Changsha, 410081 Hunan China; 7 Hunan International Joint Laboratory of Animal Intestinal Ecology and Health, Hunan Normal University, Changsha, 410081 Hunan China; 8 Hunan Provincial Key Laboratory of Animal Intestinal Function and Regulation, College of Life Science, Hunan Normal University, Changsha, 410081 Hunan China; 9 University of Chinese Academy of Sciences, Beijing, 100039 China

**Keywords:** Mitochondrial morphology, Oxidative stress, Piglets, Skeletal muscle, Taurine

## Abstract

**Background:**

Oxidative stress is a key factor that influences piglets’ health. Taurine plays an imperative role in keeping the biological system from damage. This study was conducted to investigate the protective effect of taurine against muscle injury due to the secondary effect of diquat toxicity.

**Results:**

Our study found that taurine effectively and dose-dependently alleviated the diquat toxicity induced rise of feed/gain, with a concurrent improvement of carcass lean percentage. The plasma content of taurine was considerably increased in a dose-dependent manner. Consequently, dietary taurine efficiently improved the activity of plasma antioxidant enzymes. Furthermore, taurine attenuated muscle damage by restoring mitochondrial micromorphology, suppressing protein degradation and reducing the percentage of apoptotic cells in the skeletal muscle. Taurine supplementation also suppressed the genes expression levels of the antioxidant-, mitochondrial biogenesis-, and muscle atrophy-related genes in the skeletal muscle of piglets with oxidative stress.

**Conclusions:**

These results showed that the dose of 0.60% taurine supplementation in the diet could attenuate skeletal muscle injury induced by diquat toxicity. It is suggested that taurine could be a potential nutritional intervention strategy to improve growth performance.

## Background

Oxidative stress is caused by various factors during animal growth and development, such as weaning, environmental and social challenges or vaccine injection [1], diet treatment such as a high PUFA diet [2], and excessive oxidative radicals could injure proteins and other macromolecules [3]. In the livestock industry, oxidative stress is one of the important factors that depress the growth performance of animals [4–6], causing economic loss [7]. Compelling evidence has shown that oxidative stress can trigger several diseases [8, 9]. It remains unclear whether oxidative stress depresses growth performance through the protein degradation pathway.

Some toxins and drugs also can induce muscle damage [10]. Additionally, diquat (1,1′-ethylene-2,2′-dipyridyliumdibromide monohydrate), a commonly applied bipyridyl herbicide and a potent pro-oxidant, is widely used to induce oxidative stress in different animal models [5, 11–14]. These models of oxidative stress can induce similar in increase generation of reactive oxygen species (ROS) and damages in muscle tissue.

Diquat utilizes molecular oxygen to produce superoxide anion radicals, most likely by mediating oxidative cycling without significantly changing the activity of mitochondria [12]. Diquat evokes oxidative stress in pigs, mice, and cells [4, 5, 11–17], causing damage in liver, kidney, and intestine [11, 12, 16, 17]. Recent findings support the idea that diquat can induce oxidative stress and depress animals’ growth performance [5, 11]. Moreover, it has been reported that diquat-induced oxidative stress can sustain at least 28 d following a single administration [18]. However, diquat-induce damage in skeletal muscle remains unclear.

Taurine (2-aminoethanesulphonic acid) is the most abundant free amino acid in skeletal muscle [19], exerting a protective effect in muscle damage [20]. However, mammalian’s biosynthetic ability of taurine is limited, and it uptake taurine mainly from diet [21]. Excessive taurine supplementation has adverse impacts. It depresses the growth performance of pigs with an increase in the excretion of taurine via urine [22, 23]. The urinary taurine level is significantly correlated with the creatine kinase activity of the muscle, thus it could be a potential marker to evaluate muscle damage [24]. Taurine plays a protective role against oxidative stress, which in turn affects mitochondrial biosynthesis and decreases the electron transport chain activity [25]. When exposed to chronic heat stress conditions (consistent 32 °C), oxidative stress occurs [26], and taurine exhibits alleviating effects on growth performance and protein degradation in broilers [27]. Eccentric exercise has been shown to induce oxidative stress and damage in skeletal muscle, and taurine supplementation can decrease superoxide radical production and creatine kinase levels, but not for antioxidant enzyme activity in rat muscle tissues [20, 28]. Furthermore taurine plays a protective effect in preventing exercise-induced oxidative stress in healthy young men [29].

The skeletal muscle is the most abundant tissue in the body of mammals, and its mass depends on the dynamic balance between protein degradation and synthesis; chronic stress is the potential trigger of muscle waste [30], which would decrease the mass of skeletal muscle. In livestock industry, all we want is to get more lean mass. Therefore, it is important to find certain nutritional strategies that could alleviate muscle protein degradation. Increased levels of ROS stimulate protein degradation, which is associated with skeletal muscle atrophy [31, 32]. *In vitro*, taurine appears to counteract atrophy by rescuing the majority of myotubes and mitochondria [33].

However, the underlying mechanisms of the protective effect of taurine on skeletal muscle damage have not been fully elucidated until now. Hence, in this experiment, a piglet model with diquat-induced oxidative stress was employed to study that whether diquat toxicity exerts a negative effect on skeletal muscle, the potential benefit of taurine through activating anti-oxidative defense, depressing apoptosis and protein degradation signaling pathways. Another aim was to evaluate the dose-response effects of taurine. The results of the present study might provide a nutritional strategy in oxidative stress recovery.

## Methods

### Animal experiments and diets

All procedures were allowed by the committee on animal care of the Institute of Subtropical Agriculture, the Chinese Academy of Sciences (No. ISA-2017-012). As suggested by the animal welfare protocol, all efforts were made to reduce animal suffering and use only the number of animals required to produce dependable scientific data [34]. The number of animals required is according to our previous study [16]. Thirty-five weaned piglets Duroc × (Landrace × Yorkshire) were obtained from Jiahe Agricultural Stockbreeding Corp (Changsha, China) and housed in individual pens. After 3 d acclimatization, the piglets (9.15 ± 0.14 kg), 35 days old, were randomly allotted into 5 groups (*n* = 7/treatment): (1) control piglets (CON); (2) diquat-treated piglets (DIQ); (3) piglets supplemented with 0.15% taurine and treated with diquat (LT); (4) piglets supplemented with 0.30% taurine and treated with diquat (MT);(5) piglets supplemented with 0.60% taurine and treated with diquat (HT). Diets were isoenergetic and isonitrogenic, and formulated to meet the nutritional requirement according to the National Research Council [35]. All the experimental diets were manufactured at Lifeng (Hunan) Bio-Technology Company Ltd. facilities (Changsha, China). All piglets were given free access to water and the assigned diets. Feed grade taurine (99.2% purity) was purchased from Zhangjiagang Specom Biochemical Co., Ltd. (Zhangjiagang, Jiangsu, China).

### Dosage information

Taurine was supplemented in the basic diet at the concentrations of 0.15%, 0.30% or 0.60%. The experimental diets were offered three times a day, at 07:00, 12:00 and 17:00 (total 28 d), respectively. This process was estimated to give a slight excess of supplemented diet so that the piglets have free access to designed diets. The whole experiment lasted for 28 d. The dose of taurine supplemented was based on previous studies [26]. Taurine concentration in the diets was measured by HPLC described as previously [36]. Feed composition is detailed in Table 1.

**Table 1:** Ingredient and chemical composition of the experimental diets

Items	Taurine			
	CON/DIQ	LT (0.15%)	MT (0.30%)	HT (0.60%)
Ingredient composition, %				
Corn	64.15	64.23	64.38	64.61
Soybean meal	19.80	19.40	19.00	18.10
Soybean protein concentrate	5.00	5.00	5.00	5.00
Dried whey	4.30	4.30	4.30	4.30
Fish meal	4.00	4.00	4.00	4.00
Soya bean oil	0.35	0.50	0.60	0.90
Taurine	0.00	0.15	0.30	0.60
Lysine	0.36	0.37	0.37	0.41
Methionine	0.12	0.12	0.12	0.13
Threonine	0.09	0.10	0.10	0.11
Tryptophan	0.01	0.01	0.01	0.02
CaHPO_4_	0.00	0.00	0.00	0.00
Limestone	0.52	0.52	0.52	0.52
NaCl	0.30	0.30	0.30	0.30
1% Premix^a^	1.00	1.00	1.00	1.00
Nutrient level, %				
Digestible energy, MJ/kg^b^	14.59	14.59	14.58	14.57
Crude protein	20.11	20.12	20.11	20.12
SID Lysine	1.24	1.23	1.23	1.24
SID Methionine + Cysteine	0.69	0.69	0.68	0.68
SID Threonine	0.73	0.74	0.73	0.73
SID Tryptophan	0.21	0.21	0.20	0.21
Total calcium	0.49	0.48	0.48	0.48
Total phosphorus	0.43	0.43	0.43	0.42
Digestible phosphorus	0.22	0.22	0.22	0.21
Taurine^b^	0.02	0.13	0.27	0.56

^a^Supplied per kg of diet: CuSO_4_·5H_2_O, 19.8 mg; KI, 0.20 mg; FeSO_4_·7H_2_O, 400 mg; NaSeO_3_, 0.56 mg; ZnSO_4_·7H_2_O, 359 mg; MnSO_4_·H_2_O, 10.2 mg; vitamin K (menadione), 5 mg; vitamin B_1_, 2 mg; vitamin B_2_, 15 mg; vitamin B_12_, 30 μg; vitamin A, 5400 IU; vitamin D_3_, 110 IU; vitamin E, 18 IU; choline chloride, 80 mg; antioxidants: Ethoxyquin, 20 mg

^b^Measured nutrient levels (DM basis)

### Diquat treatment

After 3 d adaptation, the challenged group of the piglets received an intraperitoneal injection of diquat (dibromide monohydrate, Sigma-Aldrich, St. Louis, MO, USA) at 9.6 mg/kg BW only a single administration, while the control group received an intraperitoneal injection of isometric 0.9% NaCl (sodium chloride, Sigma-Aldrich, St. Louis, MO, USA) solution. Diquat was dissolved in 0.9% NaCl solution to a concentration of 9.6 mg/mL and filter-sterilized (Corning Inc., Rochester, New York, USA). The injection volume was altered to enable a dose of < 10 mL per animal as described previously [5, 37]. Vomit and foam were observed in the diquat treated group but not in the control group. This appearance lasted about 1 d as the previous study [16]. All piglets were kept at room temperature at 25–28 °C and had free access to drinking water. To evaluate the effect of taurine supplementation on the growth performance of the piglets with diquat-induced oxidative stress, the piglets were weighed twice during the experiment (at the beginning and the end) and food intake was recorded, and food refusal was gathered and then weighted daily.

### Sample collection

After 28 d of feeding, all piglets fasted 12 h before sample collection. Heparinized blood samples were dram by jugular venipuncture and then centrifuged at 3,000×*g* for 15 min at 4 °C, and acquired plasma was stored at − 20 °C until analysis as described previously [6]. Piglets were electrically stunned (250 V, 0.5A, for 5–6 s) and then bled by exsanguination of precaval vein, The *longissimus dorsi* muscle and soleus were rapidly excised from the right side of the carcass. The samples were then placed in liquid nitrogen (− 196 °C) and stored at − 80 °C for RNA extraction analysis and western blot [38]. Fresh samples of *longissimus dorsi* muscle (0.5 cm^3^) were placed in 2.5% cold glutaraldehyde (Leagene Biotechnology, Beijing) for the examination of mitochondrial morphology. Urine was collected from the bladder using a disposable syringe. Total muscle (dissected free of bone, connective tissue, cartilage, and subcutaneous fat) mass of the left side of the carcass were measured. Carcass lean percentage (%) = Total muscle mass of the left side of carcass /total mass of the left side of carcass × 100%.

### Muscle protein degradation in *longissimus dorsi* and soleus muscles

To measure the rate of protein degradation, the isolated *longissimus dorsi* and the soleus muscles were incubated in the Krebs-Ringer bicarbonate buffer containing 10 mmol/L glucose under 95% O_2_–5% CO_2_ at 37 °C for 2 h after a 30 min pre-incubation at 37 °C. Tyrosine concentration was measured by the HPLC method after the derivatization of fluorescamine with a treatment of perchloric acid, heating, and fluorometry, respectively [39, 40].

### RNA extraction and real-time PCR for muscle atrophy-, mitochondria biosynthesis- and antioxidant-related genes in *longissimus dorsi*

The reverse transcription and real-time quantitative PCRs were performed as previously described [35]. Target genes mRNA expression levels were conducted by the formula 2 ^−△△Ct^ [△△Ct = (Ct _gene of interest_−Ct _GAPDH_) _treated_ − (Ct _gene of interest_−Ct _GAPDH_) _untreated_] [38]. GAPDH house-keeping gene was used as an internal control to normalize the expression of target genes. Primers for the selected genes (Table 2) were designed, using Oligo 6.0 software program.

**Table 2:** Characteristics of the primers used for real-time PCR analysis^a^

Genes	Primer (from 5’to 3′)	Size, bp	Accession No.
*CAT*	F: ACATGGTCTGGGACTTCTGG R: TCATGTGCCTGTGTCCATCT	99	XM_021081498.1
*Gpx4*	F: TGTGGTTTACGGATTCTGG R: CCTTGGGCTGGACTTTCA	181	NM_214407.1
*HSP70*	F: AGGTGCAGGTGAGCTACAAG R: CTGCGAGTCGTTGAAGTAGG	158	NM_213766.1
*SOD2*	F: CCTACGTGAACAACCTGAAC R: GATACAGCGGTCAACTTCTC	247	NM_214127.2
*TFAM*	F: AGATGCTTATAGGGCAGACTGGCA R: ACCTATGTATTGAACTGGCTGGCA	607	NM_001130211.1
*MAFbx*	F: CCAGAGAGTCGGCAAGT R: GAGGGTAGCATCGCACAAGT	374	NM_001044588
*MuRF1*	F: AGCACGAAGACGAGAAAATC R: TGCGGTTACTCAGCTCAGTC	150	NM_001184756
*GAPDH*	F: TCGGAGTGAACGGATTTGGC R: TGACAAGCTTCCCGTTCTCC	189	NM_001206359.1

^a^*CAT*, catalase; *Gpx4*, glutathione peroxidase 4; *HSP70*, heat shock protein 70; *SOD2*, superoxide dismutase 2; *TFAM*, mitochondrial transcription factor; *MAFbx,* muscle atrophy F-box; *MuRF1*, muscular ring finger protein 1; *GAPDH*, glyceraldehyde-3-phosphate dehydrogenase

### Western blotting analysis in *longissimus dorsi*

Relative protein expression levels for muscle ring finger 1 (MuRF1), muscle atrophy F-box (MAFbx) and heat shock protein 70 (HSP70) in *longissimus dorsi* muscle were determined by the Western blotting technique [35]. The resultant signals were determined using the Alpha Imager 2200 software (Alpha Innotech Corporation, San Leandro CA, USA). The primary antibodies including anti-MuRF1, MAFbx and GAPDH and the second antibodies were purchased from Proteintech Group (Chicago, IL, USA), anti-HSP70 was purchased from Abcam Group (Cambridge, England). The information for selected antibodies was shown in Table 3.

**Table 3:** Characteristics of the antibodies used for western blot analysis^a^

Antibody	Catalog number	Source	Dilution rate
HSP70	ab5439	Mouse	1:2,000
MAFbx	55,456–1-AP	Rabbit	1:600
MuRF1	12,866–1-AP	Rabbit	1:500
GAPDH	10,494–1-AP	Rabbit	1:7,000
HRP goat anti-rabbit IgG	SA00001–2	Goat	1:6,000
HRP goat anti-mouse IgG	SA00001–2	Goat	1:5,000

^a^*HSP70*, heat shock protein 70; *MAFbx,* muscle atrophy F-box; *MuRF1*, muscular ring finger protein 1; *GAPDH*, glyceraldehyde-3-phosphate dehydrogenase

### Assessment of serum antioxidant ability and inflammatory cytokines

Creatine kinase (CK; #A032), superoxide dismutase (SOD; #A001–3), blood ammonia (AMM; #A086) and blood urea nitrogen (BUN; #C013–2) were determined using the commercial kits (Nanjing Jiancheng Institute of Bioengineering, Jiangsu, China). Pig tumor necrosis factor (TNF-α; #CSB-E16980p), pig interleukin-6 (IL-6; #CSB-E06786p) were determined using ELISA kits (Cusabio, Co. Ltd., Wuhan, China). All the operations were performed following the recommended procedures.

### Taurine content in serum and urine

The content of taurine in serum and urine was measured by HPLC described as previously [36]. In brief, adding three volumes of 5% (weight/volume) sulfosalicylic acid and mixing. Acidified samples were centrifuged at 20,000×*g* for 20 min at 4 °C. The acid supernatants or standards (1–1,000 μmol/L) were diluted 1:50 in 200 mmol/L borate buffer, pH 10.4, and then analyzed by an HPLC method.

### Apoptosis detection in *longissimus dorsi*

Apoptosis cells of *longissimus dorsi* muscle were examined according to a previous study [41, 42]. In brief, terminal deoxynucleotidyl transferase-mediated deoxyurdine triphosphate nick end labeling (TUNEL) assay was performed according to the manufacture’s instruction of an *in situ* cell death detection kit (#40306ES50) (Yeasen Biotech Co., Ltd., Shanghai, China). Green granules represented positive apoptosis cells, blue granules represented nuclear staining in the muscle cells. Two sections were observed and photographed for each piglet. Ten fields (10 × 40 magnification) were randomly selected and quantification of TUNEL-positive cells was assessed using an image analyzer (Image-Pro-Plus 6.0; Media Cybernetics, MD, USA).

### Observation of transmission Electron microscopy in *longissimus dorsi*

The protocol was adapted from the previous study with some modifications [42, 43]. Briefly, the fresh *longissimus dorsi* muscle section adjacent to the 6^th^-7^th^ ribs was fixed in 2.5% glutaraldehyde (pH 7.4, 0.1 mol/L sodium cacodylate buffer) at least 2 h, then rinsing with 0.1 mol/L orthophosphoric acid (Sinopharm Chemical Reagent, Shanghai) (10 min × 3),fixed in 1% buffered osmic acid (Sinopharm Chemical Reagent, Shanghai) at least 2 h, then rinsing with 0.1 mol/L orthophosphoric acid (10 min × 3), dehydrated with grade series of acetone (Sinopharm Chemical Reagent, Shanghai) solutions at 4 °C (10 min in 50%, 70%, 90% and 100% × 2, respectively) followed by propylene oxide (Sinopharm Chemical Reagent, Shanghai), and finally embedded in Epon 812. Then, the sections were cut on ultra-microtome by using glass knives at a thickness of about 50–100 nm using Leica EM UC7 Ultramicrotome (Leica, Wetzlar, Germany). It was stained with 3% (v/v) uranyl acetate and post-staining in lead nitrate. The sections were vacuum-coated with a 5-nm layer of carbon in a high-vacuum carbon spatter coater (Q150GB, Quorum Technologies, England) and then observed with electron microscopy at an 80-kV accelerating voltage (HT7700; Hitachi, Tokyo, Japan).

### Statistical analysis

All results were expressed as mean ± SEM. The difference between [CON] vs. [DIQ] was analyzed using Student’s T-test. Data sets [DIQ] vs. [LT, MT, HT] were analyzed by One-way ANOVA followed by Duncan’s Multiple Comparison Test with SAS 8.2 statistical package (SAS Institute, Inc.). Probability values < 0.05 were considered as statistically significant. Probability values between 0.05 and 0.10 were considered to be trends.

## Results

### Growth performance and the lean percentage

All piglets survived in the present experiment. As shown in Table 4, there was no significant difference among the groups in terms of initial weight and average daily feed intake (ADFI) (*P* > 0.05). Compared with the CON group, the average daily gain (ADG) of body weight and carcass lean percentage were significantly reduced in the DIQ group (*P* < 0.05). The F/G ratio was significantly raised in DIQ groups (*P* < 0.05). Compared with DIQ group, no difference was observed in initial weight, final weight, ADFI and ADG in taurine treatment (LT, MT, and HT), the F/G ratio was significantly decreased and the carcass lean percentage was significantly increased in a dose-responsive manner (*P* < 0.05).

**Table 4:** The effect of dietary taurine on the growth performance and carcass lean percentage of the weaned pigs with diquat-induced oxidative stress*

Item	Group					*P*-value	
	CON	DIQ	TAU			*P*_D_	*P*_T_
			0.15%	0.30%	0.60%		
Initial weight, kg	9.17	9.30	9.17	9.09	9.08	0.73	0.97
Final weight, kg	22.72^A^	17.98^B^	19.55	18.69	19.72	<0.01	0.77
ADFI, kg/d	0.78	0.77	0.77	0.72	0.68	0.90	0.64
ADG, kg/d	0.50^A^	0.32^B^	0.38	0.36	0.39	<0.01	0.55
F/G	1.56^B^	2.39^Aa^	2.08^a^	2.07^a^	1.75^b^	0.01	0.04
Lean percentage, %	46.73^A^	41.15^Bc^	43.50^b^	42.84^bc^	45.64^a^	<0.01	<0.01

*Results are expressed as mean ± SEM. Values in rows with different capital letters are considered as significantly different [CON] vs. [DIQ], Values in rows with different lowercase are considered as significantly different [DIQ] vs. [LT, MT, HT]. *P*_D_: [CON] vs. [DIQ]; *P*_T_: [DIQ] vs. [LT, MT, HT]. *ADFI* Average daily feed intake, *ADG* Average daily gain, *F/G* The ratio of gain to feed intake

### Protein degradation rate

**Fig. 1:**
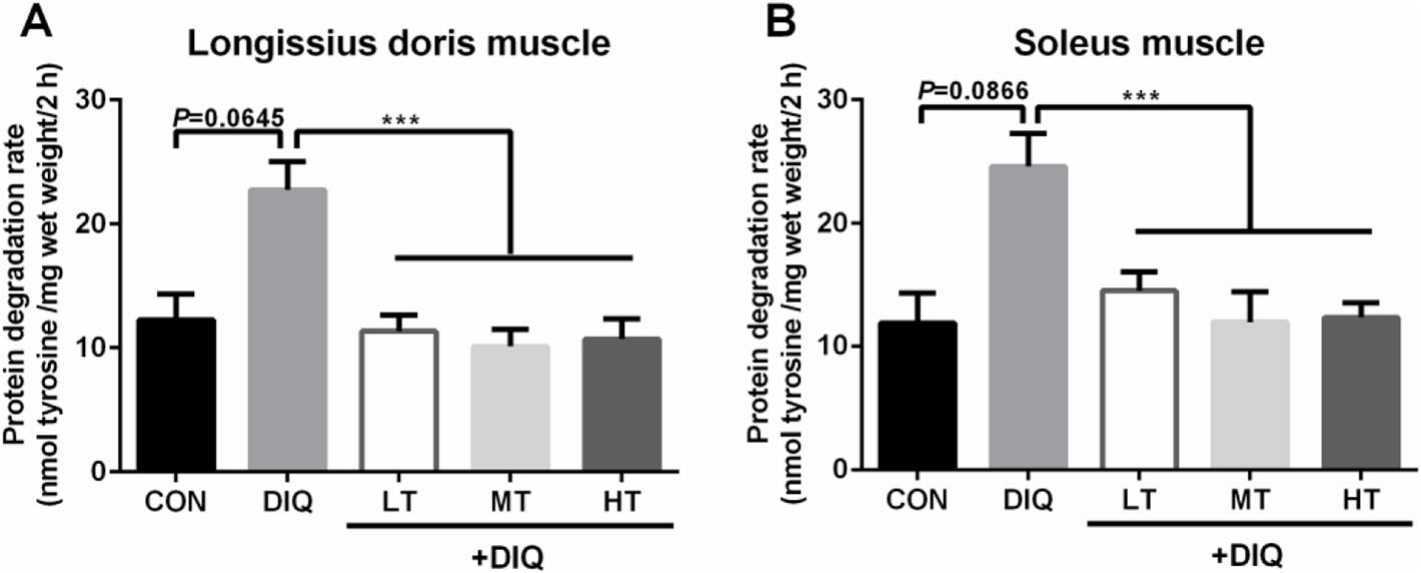
The effects of taurine supplementation on the rate of protein degradation in skeletal muscle tissue of the piglets. CON, control piglets; DIQ, diquat-treated piglets; LT, piglets supplemented with 0.15% taurine and treated with diquat; MT, piglets supplemented with 0.30% taurine and treated with diquat; HT, piglets supplemented with 0.60% taurine and treated with diquat. Results are expressed as mean ± SEM (*n* = 7). ^***^P < 0.001

To investigate whether the carcass lean percentage was correlated to protein muscle degradation and the alleviative effect of taurine, the concentration of tyrosine in the isolation buffer released from the muscle samples was measured. Compared with the CON group, diquat treatment appeared to increase the protein degradation rate in *longissimus dorsi* (*P* = 0.06) and soleus (*P* = 0.09) muscle, but the increase did not reach statistical significance (*P* < 0.10). Compared with the DIQ group, taurine treatment (LT, MT, and HT) significantly decreased the protein degradation rate in *longissimus dorsi* and soleus muscle. These results were presented in Fig. 1.

### Relative mRNA expression level of muscle atrophy-related genes and proteins

**Fig. 2:**
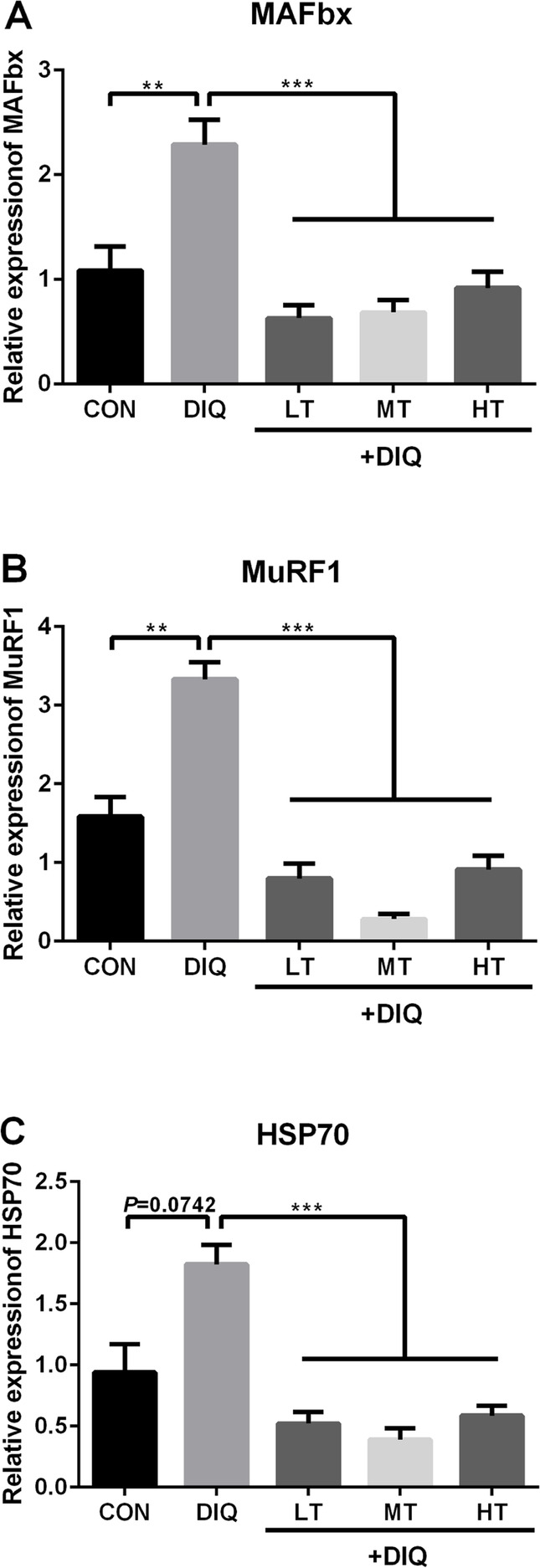
The effects of taurine supplementation on mRNA expression levels of the key genes related to protein degradation in the *longissimus dorsi* muscle of the piglets. MAFbx, muscle atrophy F-box; MuRF1, muscle ring finger 1; HSP70, heat shock protein 70. Results are expressed as mean ± SEM (*n* = 7). ^**^P < 0.01; ^***^P < 0.001

**Fig. 3:**
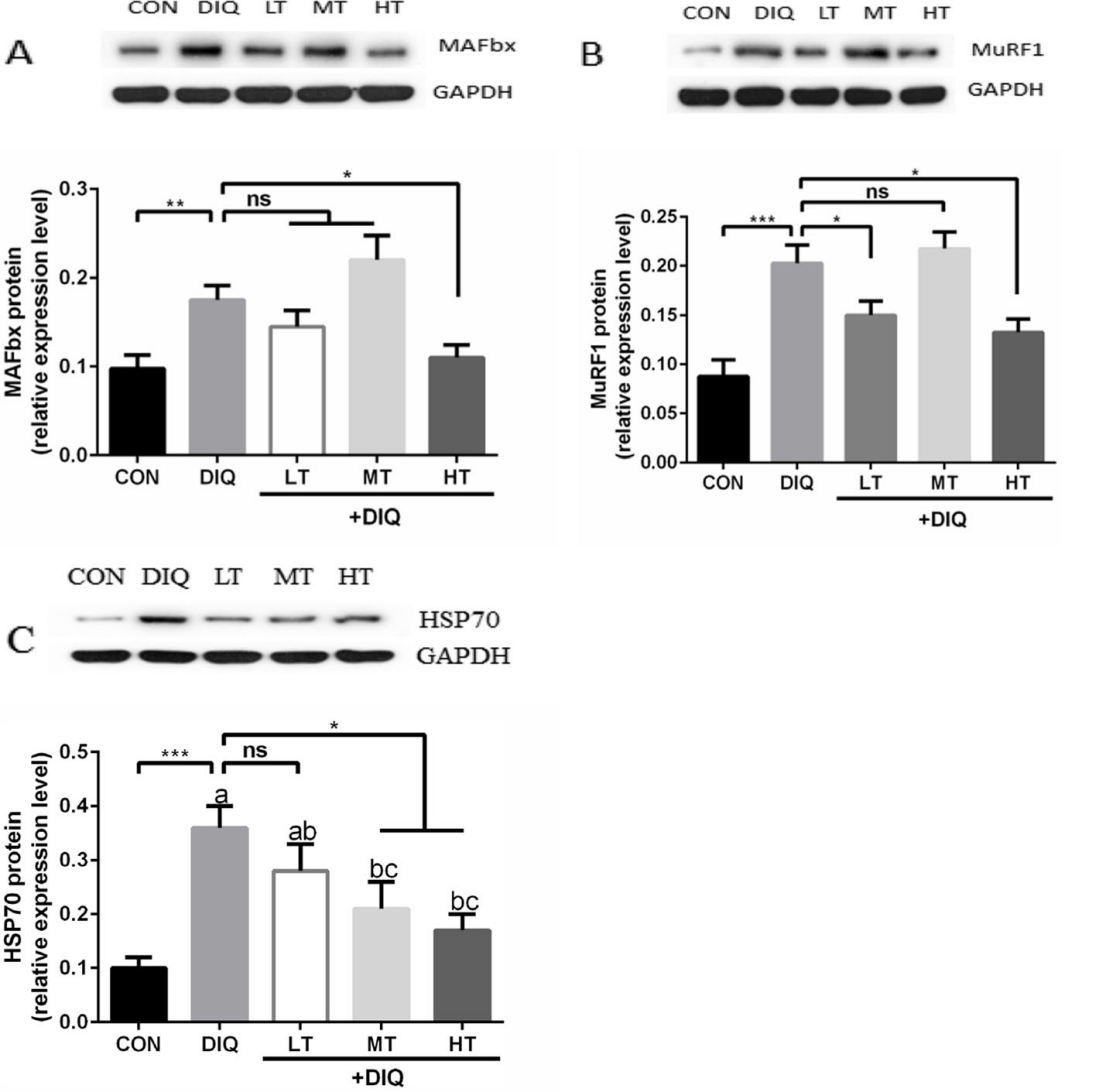
Suppressive impact of taurine on protein expression levels of MAFbx, MuRF1, and HSP70 in the *longissimus dorsi* muscle of the pigs. **a** Expression pattern of MAFbx protein in response to diquat-induced oxidative stress with 0.15%, 0.30%, and 0.60% taurine supplementation. **b** Expression pattern of MuRF1 protein in response to diquat-induced oxidative stress with 0.15%, 0.30%, and 0.60% taurine supplementation. **c** Expression pattern of HSP70 protein in response to diquat-induced oxidative stress with 0.15%, 0.30%, and 0.60% taurine supplementation. ^*^P < 0.05; ^**^P < 0.01; ^***^P < 0.001; ns, not statistically significant

To further explore the potential mechanism of protein degradation. Muscle atrophy-related key genes and proteins were examined. Compared with the CON group, the mRNA expression of MAFbx and MuRF1 were significantly up-regulated in *longissimus dorsi*, the mRNA expression of HSP70 tended to be up-regulated (*P* = 0.07), but did not reach statistical significance (*P* < 0.10). Compared with the DIQ group, taurine treatment (LT, MT, and HT) significantly decreased the mRNA expression of MAFbx, MuRF1, and HSP70 (*P* < 0.05). The mRNA expression data were presented in Fig. 2. Compared with the CON group, the protein expression of MAFbx, MuRF1, and HSP70 were significantly up-regulated in the DIQ group (*P* < 0.05). Compared with the DIQ group, HT treatment down-regulate the protein expression of MAFbx, MuRF1, and HSP70 in HT (*P* < 0.05). The protein expression data were presented in Fig. 3.

### The activity of serum antioxidant system, nitrogen metabolism and cytokines

To investigate the effect of diquat and taurine on body antioxidant system, we examined serum anti-oxidative enzyme, nitrogen metabolism, and cytokines. As shown in Table 5, compared with the CON group, the level of IL-6 and TNF-α were significantly increased in DIQ groups (*P* < 0.05). Compared with the DIQ group, the activity of SOD was significantly increased in the HT group, the concentration of IL-6 and TNF-α were significantly decreased in the HT group in a dose-dependent manner (*P* < 0.05). Compared with the CON group, the activity of CK was significantly decreased. There were no differences in AMM, BUN and CK between [CON] vs. [DIQ], [DIQ] vs. [LT, MT, and HT] (*P* > 0.05).

**Table 5:** The activity of serum anti-oxidative, oxidation product and nitrogen metabolism*

Item	Group					*P*-value	
	CON	DIQ	TAU			*P*_D_	*P*_T_
			0.15%	0.30%	0.60%		
AMM, μmol/mL	279.62	260.71	260.78	264.78	254.75	0.37	0.72
BUN, mmol/L	4.93	4.90	4.99	4.81	4.64	0.96	0.95
CK, U/mL	2.71	2.15	2.42	1.99	1.62	0.06	0.13
SOD, U/mL	32.70	33.11^ab^	28.63^b^	30.07^b^	38.27^a^	0.60	<0.01
IL-6, pg/mL	44.12^B^	114.47^Aa^	100.51^ab^	80.26^b^	52.81^c^	<0.01	<0.01
TNF-α, ng/L	18.28^B^	31.85^Aa^	17.75^b^	23.12^ab^	16.30^b^	<0.01	<0.01

*Results are expressed as mean. Values in rows with different capital letters are considered as significantly different [CON] vs. [DIQ], Values in rows with different lowercase are considered as significantly different [DIQ] vs. [LT, MT, HT]. *P*_D_: [CON] vs. [DIQ]; *P*_T_: [DIQ] vs. [LT, MT, HT]. AMM, blood ammonia; BUN, blood urea nitrogen; CK, creatine kinase; SOD, superoxide dismutase; T-AOC, total antioxidant capacity; IL-6, interleukin 6; TNF-α, tumor necrosis factor α

### Serum and urinary Taurine content

**Fig. 4:**
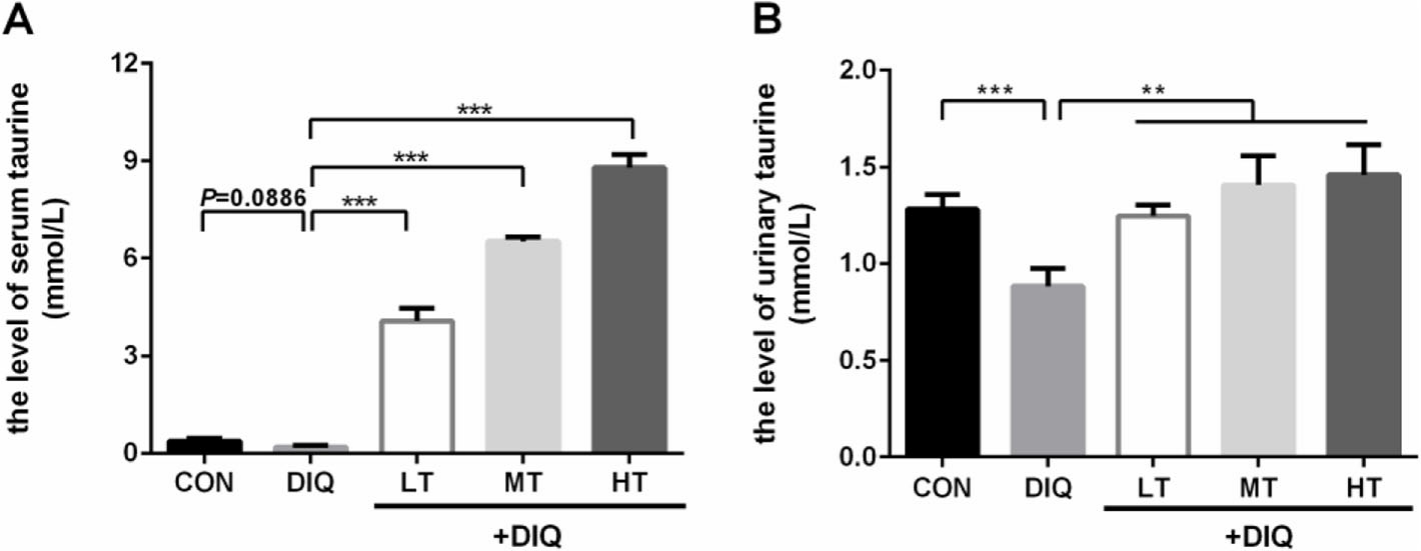
The effects of dietary taurine on serum and urinary taurine concentration of the piglets with diquat-induced oxidative stress. CON, control piglets; DIQ, diquat-treated piglets; LT, piglets supplemented with 0.15% taurine and treated with diquat; MT, piglets supplemented with 0.30% taurine and treated with diquat; HT, piglets supplemented with 0.60% taurine and treated with diquat. Results are expressed as mean ± SEM (*n* = 7).^**^P < 0.01; ^***^P < 0.001

Compared with the CON group, Serumal taurine concentration tend to decrease in the DIQ group (*P* = 0.09) but did not reach statistical significance (*P* < 0.10), diquat treatment significantly decreases the concentration of urinary taurine (*P* < 0.05). Compared with the DIQ group, taurine treatment significantly increase the concentration of taurine in a dose-dependent manner in serum (*P* < 0.05), and taurine treatment (LT, MT, and HT) significantly increases the concentration of urinary taurine (*P* < 0.05). These results were presented in Fig. 4.

### Skeletal muscle cell apoptosis

**Fig. 5:**
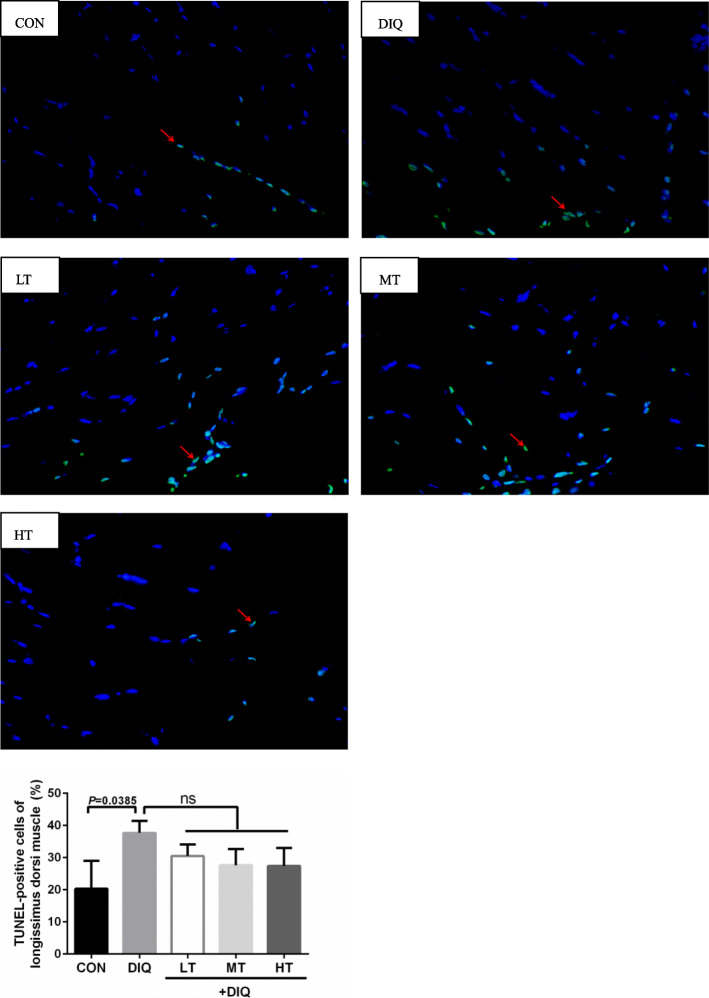
The effect of taurine supplementation on the percentage of apoptotic cells in the *longissimus dorsi* muscle of the pigs. CON, control piglet; DIQ, diquat-treated piglets; LT, piglets supplemented with 0.15% taurine and treated with diquat; MT, piglets supplemented with 0.30% taurine and treated with diquat; HT, piglets supplemented with 0.60% taurine and treated with diquat. ^a)^ Results are expressed as mean ± SEM. ns, not statistically significant

As shown in Fig. 5, compared with the CON group, toxic effects of diquat significantly increase the percentage of apoptotic cells (*P* < 0.05). Compared with the DIQ group, taurine treatment decreased the percentage of apoptotic cells in numerical (*P* > 0.05).

### Mitochondrial morphology

**Fig. 6:**
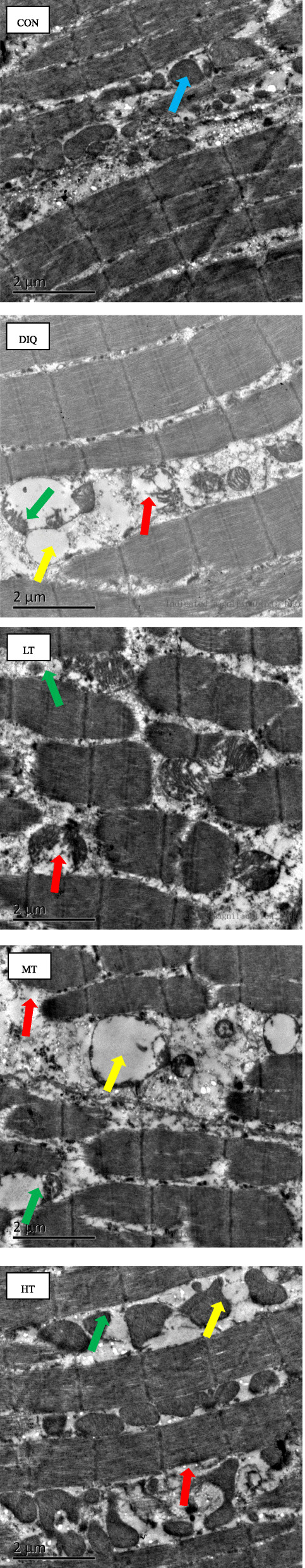
The effects of taurine supplementation on the morphology of mitochondria in the *longissimus dorsi* muscle of the piglets. CON, control piglets; DIQ, diquat-treated piglets; LT, piglets supplemented with 0.15% taurine and treated with diquat; MT, piglets supplemented with 0.30% taurine and treated with diquat; HT, piglets supplemented with 0.60% taurine and treated with diquat. Blue arrows point to the well-developed mitochondria. Red arrows point to the membrane-faulted mitochondria. Yellow arrows point to the vacuolated and expanded mitochondria. Green arrows point to the membrane-dissolved mitochondria

To investigate the effect of diquat and taurine on muscular mitochondrial morphology, TEM was performed. As shown in Fig. 6, in the CON piglets, well-developed mitochondria with preserved membranes and cristae were observed. In contrast, swollen and irregular-shaped mitochondria with disrupted and poorly defined cristae were observed in the DIQ groups. In the HT group, mitochondria of the muscle tissue showed a clearer defined membrane structure.

### Relative mRNA expression level of the antioxidant- and mitochondria biosynthesis-relatedd genes

**Fig. 7:**
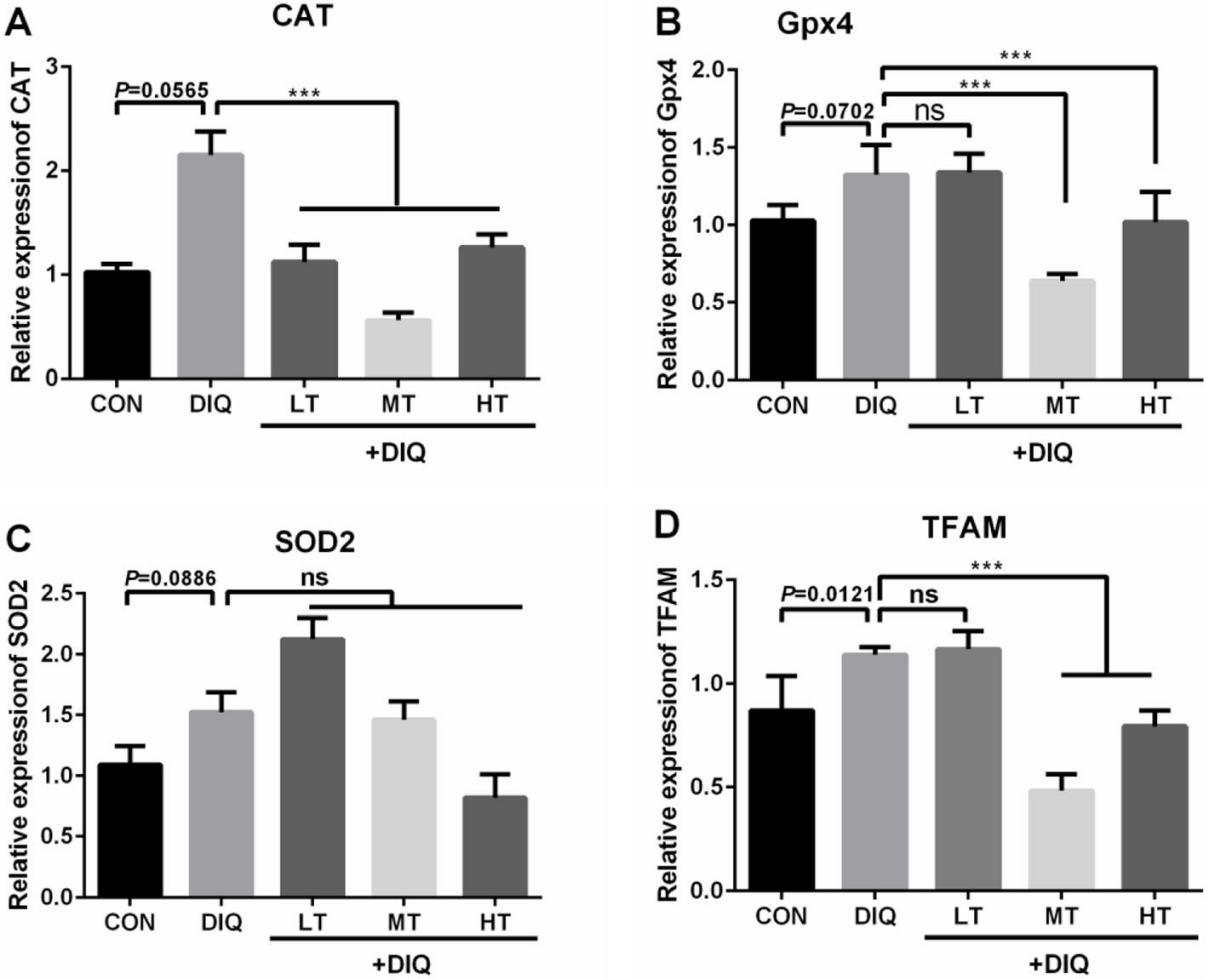
The effects of taurine supplementation on mRNA expression levels of the key genes related to antioxidant and mitochondrial biogenesis in the *longissimus dorsi* muscle of the pigs. CAT, catalase; Gpx4, glutathione peroxidase 4; SOD2, superoxide dismutase 2; TFAM, mitochondrial transcription factor A. Results are expressed as mean ± SEM (*n* = 7). ^***^P < 0.001; ns, not statistically significant

To further determine the potential mechanism of the antioxidant effect of taurine, key genes of antioxidant and mitochondria biosynthesis were detected in *longissimus dorsi*. Compared with CON group, the expression of genes [*CAT* (*P* = 0.0565), *Gpx4*(*P* = 0.0702) and *SOD2* (*P* = 0.09)] related to antioxidant activity appear to up-regulated in the DIQ group, but did not reach statistical significance (*P* < 0.10), the mRNA expression of TFAM was significantly increased in DIQ group (*P* < 0.05). Compared with the DIQ group, the expression of *CAT*, *Gpx4* and *TFAM* were significantly decreased in MT and HT group (*P* < 0.05). These results were presented in Fig. 7.

## Discussion

The present study demonstrated that high dietary taurine content (0.60%) resulted in several beneficial effects, such as improving anti-oxidation ability and inhibiting the degradation of skeletal muscle protein responding to diquat induced oxidative stress. These findings suggested that supplementation of taurine could alleviate oxidative stress-induced muscle injuries.

Diquat is known to induce free radical production through redox cycling, and then impair the redox balance; therefore, pigs divert nutrients from growth to anti-oxidative response, consequently slowing down the growth rate and development [11, 12]. Our results are consistent with the findings of previous studies, showing that diquat-induced oxidative stress restricts animal growth and development [5, 11, 14, 41, 44]. Taurine is abundant in skeletal tissues and is involved in various biological functions, such as anti-oxidation and muscle growth [19]. As expected, in the present study, we found that dietary taurine supplementation (0.60%) could alleviate the decrease in growth performance and carcass percentage of muscle mass in the piglets with diquat-induced oxidative stress.

Antioxidant enzymes form the first line of defense against oxidative stress in an organism, and the activity of the enzymes mainly depends on the oxidative equilibrium of cells. Therefore, we examined the redox state of the body and evaluated the effect of dietary taurine supplementation on the key enzyme activity of plasma. It has been reported that the oxidative condition of a cell is the most important factor modulating the expression of the genes related to antioxidant enzymes [45]. The cytokine TNF-α upregulates the mRNA expression level of *SOD2* (a key mitochondrial antioxidant enzyme) [46],and a previous study has proved that *SOD2* upregulation is the signal enabling cell survival [47]. When subjected to pro-oxidant paraquat, the transcript levels of *Gpx*, *SOD2*, and *CAT* were increased [45]. Interestingly, in the present study, we observed a downregulation of the mRNA expression level of these key genes after 0.60% taurine supplementation. In high fat-induced oxidative stress conditions, the plasma taurine content is decreased [48, 49]. Accordingly, our results showed that diquat-induced oxidative stress tended to decrease the content of plasma taurine, suggesting that taurine might be a biomarker of oxidative stress status. As reported previously, taurine supplementation significantly increased its content in the plasma [20]. Our results showed that the plasma content of taurine increased in a dose-dependent manner (Fig. 4a). The changes in taurine content in skeletal muscles have been invested in our previous study, and the value increased in a dose-dependent manner while dietary taurine increased in soleus muscle [16].

Excessive ROS leads to mitochondrial dysfunction, decreases membrane potential of mitochondria and downregulates the expression level of genes related to mitochondrial biogenesis [50–52]. Mitochondrial transcription factor A protects the mitochondrial DNA from ROS and degradation, while enhancing mitochondrial function [32]. Long-term supplementation of taurine reduces mitochondrial ROS production, alleviates mitochondria protein oxidation status, and reduces mitochondrial structural damage including inhibition of mitochondrial swelling [53, 54], inhibits mitochondria-mediated apoptosis [52]. Our results showed that 0.60% taurine supplementation could restore the morphology of mitochondria, consistenting with previous findings [55, 56]. During the periods of excessive supplementation of amino acids, and urinary amino acids excretion is increased [57]. This has been proved with taurine supplementation as well [23]. The content of urinary taurine was significantly decreased in the DIQ group compared with that in the CON group. Basal plasma taurine level is relatively low, 10–100 μmol/L in humans and 650–770 μmol/L in mice [58]. In weaned piglets, the level of taurine is at 11.9–50.1 μmol/L [59, 60]. In our study, the basal taurine level was at about 0.38 mmol/L, suggesting that our results were reliable. These observations indicate that 0.60% taurine in the diet can restore muscle injury and ameliorate mitochondrial dysfunction induced by oxidative stress.

Muscle protein degradation occurs due to oxidative damage, nutrition starvation, and burn injury [31, 35, 40, 42, 61]. Several studies have evaluated the total muscle protein degradation by examing the rate of tyrosine release from the isolated muscle, as tyrosine is not metabolized in muscle cells [39]. To investigate whether taurine could alleviate protein degradation in skeletal muscle, the concentration of tyrosine in the isolation buffer released from the muscle samples was measured. Interestingly, we found that taurine could inhibit the protein degradation of the *longissuim dorsi* muscle and soleus muscle tissues. Using the TUNEL analysis method to detect muscle cell apoptosis, and we further confirmed that the beneficial effect of tauirne on muscular atrophy of diquat-injected piglets. Previous research also validates these results [41]. Chaperones, in particular, HSP70 contribute to key steps in protein degradation via the ubiquitin-proteasome system [62]. We then examined the relative protein expression level of HSP70 and found that oxidative stress-induced HSP70 protein increased while taurine supplementation could down-regulate its expression level. Increased content of ROS stimulates protein degradation, which is associated with skeletal muscle atrophy [31, 32]. The ubiquitin-proteasome pathway is considered one of the main pathways of protein degradation, and as muscle-specific ubiquitin ligases, MuRF1 and MAFbx play an important role in muscle degradation and muscle atrophy [35, 40, 42]. Under oxidative stress conditions, the system is critical to ensure proteostasis and cell survival [63]. Taurine supplementation can alleviate chronic heat stress-induced increase in the mRNA expression level of *MuRF1* and *MAFbx* [27]. It has also been reported that increasing dietary taurine content is a good strategy to decrease the severity of dystropathology [64]. Our results are consistent with this research. The changes in the protein expression level of MAFbx and MuRF1 were correlated with the variation trend of the final weight of the pigs, indicating that 0.60% taurine supplementation was superior to 0.15% or 0.30% taurine supplementation. It is suggested that 0.60% dietary taurine supplementation might be a promising anti atrophy strategy for improving animal health and life quality. However, there still have some limitations, for we did not explore the possible mechanism on the cellular level. It would be of great interest to our following study.

## Conclusions

In summary, our study partially revealed that acute diquat toxicity caused a serious dysfunction in skeletal muscle of weaned piglets. Taurine supplementation could alleviate the growth performance suppress and lean mass percentage decrease, also restore the mitochondrial morphology and inhibit protein degradation of the muscle tissue. We speculated that dietary taurine could attenuate the oxidative stress toxicity of diquat mainly by regulating the antioxidant enzyme activity, the expression levels of the key genes, and inhibiting the ubiquitin-proteasome pathway. Taurine would be an available nutritional strategy to improve animal and human health.

## Data Availability

The data used to support the findings of this study are available from the corresponding author upon reasonable request.

## Abbreviations

ADG: Average daily gain
ADFI: Average daily feed intake
BUN: Blood urea nitrogen
CAT: Catalase
BW: Body weight
CK: Creatine kinase
Gpx4: Glutathione peroxidase 4
HSP70: Heat shock protein 70
IL-6: Interleukin-6
MAFbx: Muscle atrophy F-box
MuRF1: Muscle ring finger 1
ROS: Reactive oxidative species
PUFA: Polyunsaturated fatty acid
SOD2: Superoxide dismutase 2
TEM: Transmission electron microscopy
TFAM: Mitochondrial transcription factor A
TNF-α: Tumor necrosis factor α
